# Clinical outcomes with paliperidone palmitate 3-monthly injection as monotherapy: observational 3-year follow-up of patients with schizophrenia

**DOI:** 10.1192/j.eurpsy.2024.13

**Published:** 2024-03-07

**Authors:** Ivana Clark, Phoebe Wallman, Siobhan Gee, David Taylor

**Affiliations:** 1 Pharmacy Department, Maudsley Hospital, South London and Maudsley NHS Foundation Trust, Denmark hill London SE5 8AZ, UK; 2 Institute of Pharmaceutical Science, King’s College London, 150 Stamford Street, London SE1 9NH, UK; 3 Institute of Psychiatry, Psychology & Neuroscience, King’s College London, 16 De Crespigny Park, London SE5 8AF

**Keywords:** continuation, long-acting, paliperidone 3-month, relapse, schizophrenia

## Abstract

**Background:**

Paliperidone palmitate 3-monthly (PP3M) has been tested in 1-year controlled studies. The aim of this study was to examine the relapse outcomes with PP3M monotherapy at 3 years in patients with schizophrenia.

**Methods:**

This was an observational, non-interventional study of patients started on PP3M according to their clinical need. All patients had a diagnosis of schizophrenia (ICD-10 F20) and were between 18 and 65 years of age. The study took place in a mental health facility in South East London, UK.

**Results:**

Among the 166 patients who started PP3M, 97 (58%) met inclusion criteria and were observed for 36 months. In total, five patients (5%) experienced a relapse (defined as step-up in clinical care) while on PP3M. There were no relapses between months 18 and 36. Of the original 97 patients, 56 (58%) remained on PP3M monotherapy at 3 years, and 71 (73%) remained on either PP3M or paliperidone palmitate one-monthly. Reasons for discontinuation of PP3M included patient refusal (n = 11, 33% of discontinuations) and adverse effects in (n = 8, 24%).

**Conclusion:**

PP3M is a highly effective monotherapy treatment for reducing relapse in people with schizophrenia.

## Introduction

Schizophrenia is a chronic condition often requiring life-long treatment. Long-acting antipsychotics (LAIs) are more effective than oral medication in preventing relapse and improving long-term clinical outcomes [1–4]. In recent years, very LAI formulations have been introduced [5–11], but little is known of their utility in clinical practice. Paliperidone palmitate is the only LAI available as a three-monthly and six-monthly formulation. The paliperidone palmitate 3-monthly (PP3M) formulation is designed to be given four times a year to patients diagnosed with schizophrenia who have previously been stabilised with one-monthly paliperidone (PP1M) [12]. In addition to its less frequent injections and improved medication adherence, PP3M is generally well tolerated and shares a similar tolerability profile with on PP1M [13]. We have previously reported 2-year continuation and relapse rates with PP3M in patients with schizophrenia [14, 15]. Now, we report these outcomes at 36 months of follow-up.

## Methods

This study was approved by the Drug and Therapeutics Committee at South London and Maudsley NHS Foundation Trust (approval codes: SLAM/DTC/2016/3 and DTC/2021/122), which oversees medicines use in the trust. This was a non-interventional, observational, and retrospective study on looking at patients diagnosed with schizophrenia prescribed PP3M antipsychotic monotherapy initiated between November 2016 and September 2018 (a sub-analysis of patients on antipsychotic polytherapy is available in the Supplementary material). Patients were prescribed PP3M based on their individual clinical needs after having at least four maintenance doses of PP1M. Antipsychotic monotherapy was defined as the regular prescription of PP3M in the absence of any other prescription of any sort (e.g. regular or “when necessary”) any medication licensed as an antipsychotic. Patient data were gathered using the trust’s electronic patient health records. Only pharmacy staff actively engaged in patient care had access to the anonymised data, which were securely stored on the trust’s database system.

The primary outcomes were continuation with PP3M monotherapy or relapse. The patient group observed in our initial paper was followed up for additional 12 months [14]. The original study registered patients initiated on PP3M before September 2018. Excluded were patients who were over 65 years of age and those without an F20 diagnosis (ICD-10) (see original paper for full details on Methods) [14]. The patient data collected were age, sex, ethnicity, care setting on initiation of paliperidone palmitate long-acting injections (PPLAIs), unresponsiveness to treatment (defined as previous consideration or actual treatment with clozapine), substance abuse (SA; history of SA [defined as any mention of SA in health records at any time] or confirmed ICD-10 co-diagnosis [F10-F19]), co-prescription of another antipsychotic for at least 1 month, prior PP1M dose and treatment duration, initial PP3M dose, and any dose change. Electronic patient health records were consulted to collect information on outcomes (treatment continuation with PP3M or relapse during the follow-up period), reasons for discontinuation, and next antipsychotic treatment (prescribed within 6 months of PP3M discontinuation).

If PP3M was discontinued, for the purposes of the study, the discontinuation date was recorded as the date 4 calendar months after the last administered dose or as the date of death if this was sooner. Relapse was defined as escalation of clinical care such as the referral to community mental health crisis team, seeking psychiatric care through accident and emergency services (A&E) due to deterioration in mental state, or direct admission to psychiatric hospital.

Secondary outcomes were continuation and relapse for all PPLAI formulations including switching to PP1M after PP3M treatment cessation. PP1M discontinuation date was recorded as 1 month after last injection administration. Reasons for discontinuation of PPLAIs were also recorded.

### Statistical analysis

Descriptive statistics were used to compile baseline characteristics, with continuous data presented as means and standard deviations. Categorical data were reported as frequencies and percentages. Kaplan–Meier plots were generated to present time to discontinuation and time to relapse. The statistical analysis was conducted using R version 4.0.2 (R Foundation for Statistical Computing, Vienna, Austria). We estimated relative risk (RR) of continuation and relapse in people with a history or diagnosis of SA using Georgiev’s calculator [16].

## Results

### Inclusion criteria

**Figure 1.**
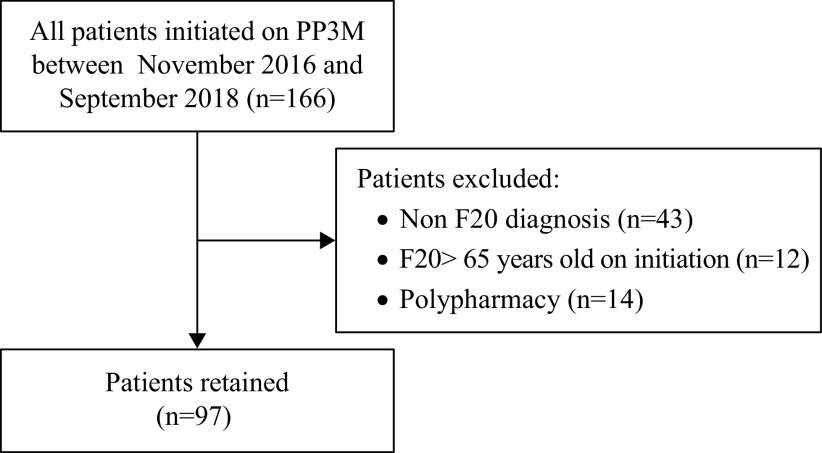
Inclusion criteria.

Figure 1 shows the process of enrolment for this study. Out of 166 patients initiated on PP3M before September 2018, 97 patients (58%) met inclusion criteria and were followed up for 36 months. Sub-analysis of 111 patients (including 14 patients who were on antipsychotics at some time during PP3M treatment) can be found in the Supplementary material.

### Baseline characteristics

In total, 97 patients were included in this analysis. Table 1 provides a summary of their demographic and clinical information. Information on PP3M initial dose and dose changes is summarised in Table 2. The ethnicity information for patients who experienced relapse (n = 5) and those who discontinued treatment (n = 41) is provided in Table 3.

**Table 1.** Baseline characteristics

Characteristic	Total	
	*N* = 97	
*Age (years)*		
Mean, SD (range)	43.5, 11.1 (20–65)	
*Prior duration of PP1M (months)*		
Mean, SD (range)	33.7, 24.8 (3–85)	
	*n* (%)	
*Sex*		
Female	28	(28.9)
Male	69	(71.1)
*Ethnicity*		
Asian/Asian British (*Indian subcontinent)*	6	(6.2)
Black/Black British	65	(67.0)
Mixed background	5	(5.2)
Other	7	(7.2)
White	14	(14.4)
*Care setting on initiation of PPLAI*		
Inpatient	50	(51.5)
Outpatient	47	(48.5)
*Considered treatment-responsive*		
Yes	69	(71.1)
No	28	(28.9)
*Substance abuse*		
No	37	(38.1)
History	51	(52.6)
Diagnosis	9	(9.3)
F10	1	(1)
F12	5	(5.2)
F19	3	(3.1)
*Prior PP1M dose*		
150 mg	30	(30.9)
100 mg	42	(43.3)
75 mg	15	(15.5)
50 mg	10	(10.3)

**Table 2.** PP3M characteristics

PP3M	Total	
	*N* = 97	
*Initial PP3M dose* (*n* [%])		
525 mg	28	(28.9)
350 mg	42	(43.3)
263 mg	16	(16.5)
175 mg	11	(11.3)
*Dose change* (*n* [%])		
None	83	(85.6)
Increase (*n* [%])	3	(3.1)
Worsening symptoms	2	(66.7)
Patient request	1	(33.3)
Decrease (*n* [%])	11	(11.3)
Patient request	5	(45.5)
Adverse effects	4	(36.4)
Improved condition	1	(9.1)
Unclear	1	(9.1)

**Table 3.** The ethnicity of patients who discontinued treatment or relapsed during the observational period

Ethnicity – *Discontinuers*	Total	
	*n* = 41	
Asian/Asian British (*Indian subcontinent)*	2	(5)
Black/Black British	30	(73)
Mixed background	2	(5)
Other	3	(7)
White	4	(10)
Ethnicity – R*elapse patients*	Total	
	*n* = 5	
Black/Black British	3	(60)
Other	1	(20)
White	1	(20)

## Substance abuse

Of the total cohort of 97 patients, 51 (52.6%) had a history of SA and 9 patients (9.3%) had a confirmed ICD-10 co-diagnosis (cannabis-related disorder [n = 5], mental and behavioural disorders due to multiple drug use and use of other psychoactive substances [n = 3], mental and behavioural disorders due to use of alcohol [n = 1]). In all, 73% of discontinuers and 54% of continuers had a history of SA or confirmed ICD-1O diagnosis. A history or diagnosis of SA was not associated with risk of discontinuation (RR = 1.35; 95% CI 0.99–1.87) or risk of relapse (RR = 1.31, 95% CI 0.82–2.10).

## Continuation

**Figure 2.**
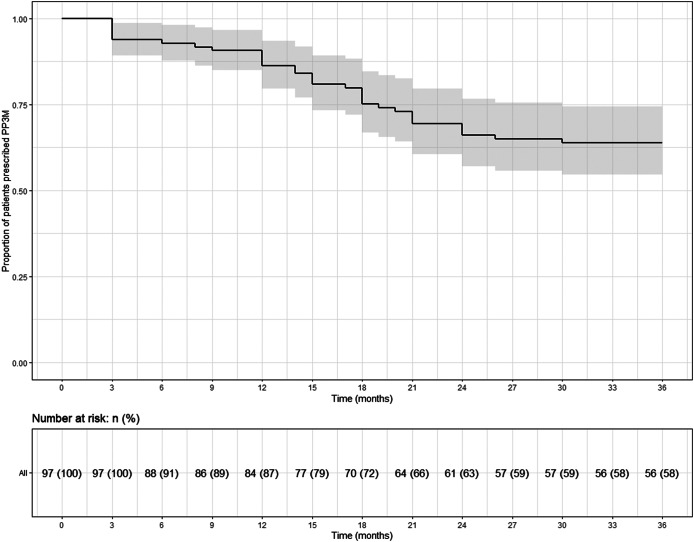
Kaplan–Meier plot showing the proportion of patients prescribed paliperidone palmitate 3-monthly (PP3M) monotherapy since initiation and at 36 months.

**Figure 3.**
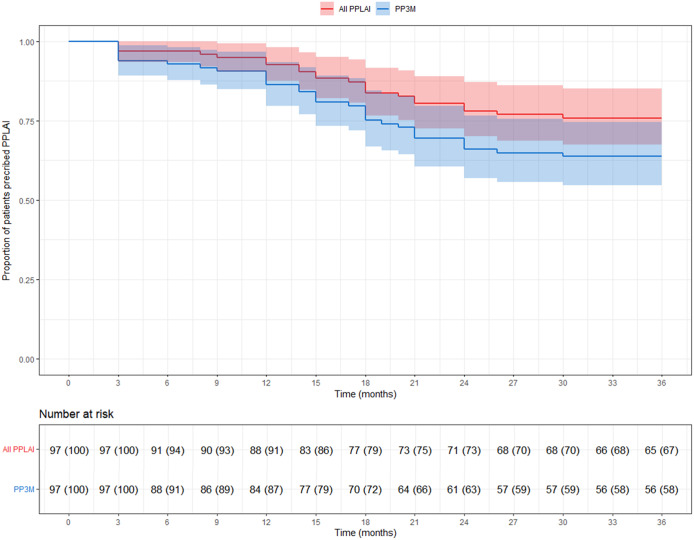
Kaplan–Meier plot showing the proportion of patients (with 95% confidence interval) prescribed paliperidone palmitate long-acting injection (PPLAI) since paliperidone palmitate 3-monthly (PP3M) initiation. All PPLAIs (PP3M followed by PP1M and in some cases PP3M again) are shown in red and PP3M only is shown in blue.

Of the total cohort of 97 patients who started PP3M, 56 (58%) remained on PP3M monotherapy at 36 months (Figure 2). Of the 41 patients who stopped PP3M, 2 died and 6 were lost to follow-up. Of the remaining 33 patients who discontinued PP3M, 15 (46%) received PP1M instead (Table 4). In total, 71 patients (73%) continued a formulation of PPLAI (1M or 3M) for the entire study period or switched to 1M within 6 months of discontinuing 3M. The overall continuation rate for all PPLAI formulations (1M and 3M) within the same timeframe was 67% (Figure 3). Tables 4 and 5 present the reasons for discontinuation. We noted and recorded the medication prescribed after PP3M discontinuation (Table 4). Patients receiving no medication for at least 6 months after cessation were recorded as switching to “no medication.”

**Table 4.** Discontinuation from PP3M

Clinical outcome after 3 years (PP3M)	*N* = 97	
*Outcome 3 years after initiating PP3M* (*n* [%])		
Continuation	56	(57.7)
Discontinuation	33	(34.0)
Attrition	8	(8.2)
	*n* = 33	
*Reasons for discontinuation* (*n* [%])		
Patient refusal	11	(33.3)
Adverse effects	8	(24.2)
Perceived inefficacy	7	(21.2)
Patient request	4	(12.1)
Independent health condition^a^	2	(6.1)
Need more flexible dose adjustment	1	(3.0)
*Next medication (6 months after last depot)* (*n* [%])		
PP1M	15	(45.5)
No medication^b^	7	(21.2)
Risperidone (oral)	4	(12.1)
Aripiprazole (oral)	3	(9.1)
Haloperidol (oral)	1	(3.0)
Quetiapine (oral)	1	(3.0)
Zuclopenthixol (LAI)	1	(3.0)
Lurasidone (oral)	1	(3.0)
	*n* = 8	
*Reasons for attrition* (*n* [%])		
Lost to follow-up^c^	6	(75)
Died^d^	2	(25)

a Independent health condition: cancer (*n* = 1) and kidney problems (*n* = 1).

b All refused medication.

c Lost to follow-up: missing person (*n* = 3), left country (*n* = 1), changed trust (*n* = 1), disengagement (*n* = 1).

d Deaths classified using trust’s electronic patient care records: non-adherence to diabetes medication (*n* = 1) and unknown (*n* = 1).

**Table 5.** Discontinuation from PPLAI (1M or 3M)

Clinical outcome after 3 years (PPLAI)	*n* = 97	
*Outcome 3 years after initiating PPLAI* (*n* [%])		
Continuation	65	(67.0)
Discontinuation	22	(22.7)
Attrition	10	(10.3)
	*n* = 22	
*Reasons for discontinuation* (*n* [%])		
Patient refusal	10	(45.5)
Adverse effects	5	(22.7)
Patient request	4	(18.2)
Perceived inefficacy	2	(9.1)
Independent health condition	1	(4.5)
	*n* = 10	
*Reasons for attrition* (*n* [%])		
Lost to follow-up^a^	8	(80.0)
Died	2	(20.0)

a The two additional patients lost to follow-up were because of changing trust and being discharged to their GP.

The most frequently recorded reason for discontinuation was patient refusal (33% of discontinuers). Adverse effects were the primary reason for PP3M discontinuation in eight patients (24% of discontinuers). Individual adverse effects responsible for discontinuation were hyperprolactinaemia (n = 3), weight gain (n = 4), extrapyramidal movement disorder (n = 1), anorgasmia (n = 1), erectile dysfunction (n = 1), anxiety (n = 1), and insomnia (n = 1) (some patients gave more than one reason).

## Relapse

**Figure 4.**
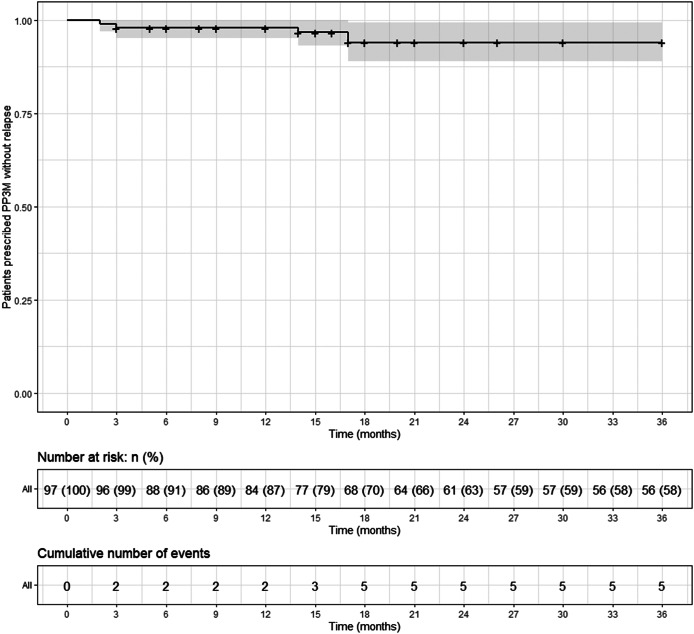
Kaplan–Meier plot showing the proportion of patients that relapsed (with 95% confidence interval) while being prescribed paliperidone palmitate 3-monthly (PP3M) monotherapy. Patients who discontinued were censored (shown as a dash on the plot).

**Figure 5.**
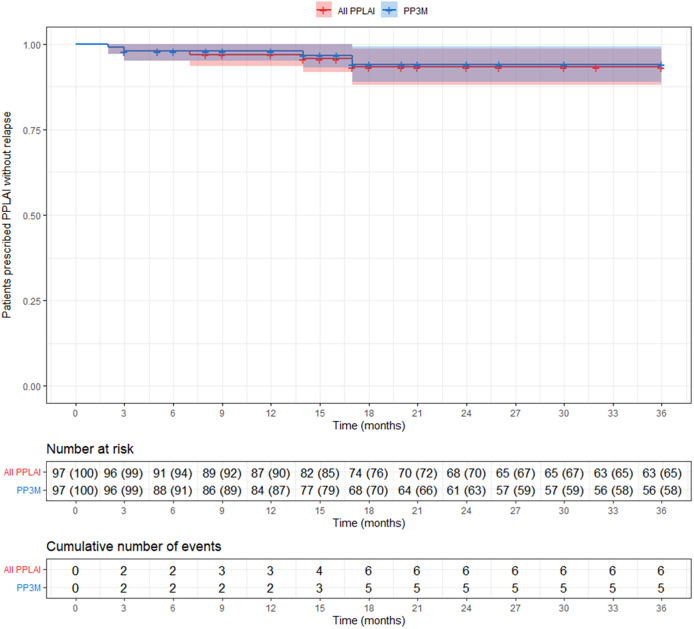
Kaplan–Meier plot showing the proportion of patients that relapsed (with 95% confidence interval) while being prescribed paliperidone palmitate long-acting injection (PPLAI). Patients who discontinued were censored (shown as a dash on the plot). All PPLAI (paliperidone palmitate 3-monthly [PP3M] monotherapy followed by paliperidone palmitate one-monthly [PP1M] and in some cases PP3M) is shown in red and PP3M only is shown in blue.

Overall, five patients (5%) relapsed on PP3M during the 36 months observational period (Figure 4). No patient on PP3M relapsed between 18 and 36 months. Including all those on PPLAI (1M and 3M), six patients (6%) experienced a relapse episode (Figure 5), all of them within the first 18 months.

## Discussion

In this study of 97 patients initiated on PP3M monotherapy and followed up for 36 months, 58% were still on treatment with PP3M at the end of the observational period. The relapse rate over the 3 years was 5%, with no relapses occurring between months 18 and 36. In terms of overall PPLAI use (PP1M and PP3M), 67% of patients continued on PPLAIs at 3 years and 6% experienced relapse over the study period.

In this study, no one relapsed after 18 months of PPLAI treatment, including the patients on antipsychotic polypharmacy (see Supplementary material). This is an extremely important finding. It indicates that relapse prevention is possible if the dose and frequency of antipsychotic are optimised in fully compliant patients who are confirmed responders to that medication. Currently, it is assumed that noncompliance is just one precipitant to relapse and that other factors (e.g., SA) can bring about relapse even in compliant patients [17]. The majority of our cohort had either abused substances in the past or had a confirmed SA diagnosis. The overall rates of discontinuation and relapse for all patients on PPLAIs, including those with a history or diagnosis of SA, remained very low.

Our findings present the possibility that most or all relapse in schizophrenia is at least partly the result of suboptimal dosing (and, in turn, presumably subtherapeutic antipsychotic plasma levels). There is emerging evidence that supports the concept that all or most relapse is treatment-related. In a case–control study of schizophrenia patients on PP1M conducted in our trust in 2021 [18], we found an association between relapse and longer intervals between PP1M doses and a lower number of PP1M injections administered over a 12-month period. Relapse was very uncommon in those receiving 12 injections per year. In addition to this, a retrospective 24-month comparative study from Spain found that 88% of patients on PP3M did not experience any relapse after 18 months of treatment [19]. The same study reported a two-fold significantly lower risk of hospital admissions and a significantly lower number of A&E visits for PP3M in comparison with PP1M, aripiprazole depot, and oral antipsychotics [19]. This advantage is assumed to reflect better maintenance of therapeutic plasma levels in PP3M patients, presumably because the proportion of PP3M patients receiving four doses a year is higher that the proportion receiving 12 doses a year of PP1M (as our studies of these two formulations have shown).

There are few studies of PP3M of similar methodology and duration to this study. Reported continuation rates ranged from 66% [20] to 96% [21] at 12 months and 52.5% [20] at 24 months. Relapse rates were reported as 18% [17, 20] at 12 months and varied from 4% [22] to 21.4% [20] at 24 months. These findings are broadly similar to those reported here.

Continuation rates with other shorter-acting LAIs are generally lower than those reported here. Monthly aripiprazole (ALAI) has been reported to have continuation rates of 94% at 6 months [23] to 59% at 12 months [24]. For the Consta formulation of risperidone LAI reported continuation rates were 58% at 12 months [25], 85% at 24 months [26], and 16% at 36 months [27]. In the present cohort, 91% were on PP3M at month 6, 87% at 12 months, and 63% at 24 months. Continuation rates with PP3M are better than those reported for shorter-acting LAIs, but it should be noted that patients receiving PP3M were previously stabilised on PP1M for at least 6 months prior to switching to PP3M. Set against this, however, is the fact that in our study, the average prior treatment duration with PP1M was 33 months, effectively extending the observation period for our reported outcomes to over 5 years.

In this study, black patients made up the majority (67%) of our study sample. This proportion is vastly different from our local population where approximately 20% are black African or Afro-Caribbean. Black people may have higher rates of schizophrenia diagnosis [28, 29] but not to the extent that could explain this difference. So, black people are overrepresented in our observational cohort, although the reason for this remains unclear. Previous studies in our trust have found no ethnicity-related differences in prescribing practice [30–32]. The over representation of black people in our observational cohort has implications for generalisability of our findings to other ethnic groups.

For both PP3M and PPLAIs (1M and 3M), patient refusal was the primary reason for discontinuation. The same phenomenon has been reported in other LAI observational studies [24, 26, 27]. We could not identify the precise reasons given by patients (if any) for refusing to continue although some obviously preferred treatment with PP1M. The patients’ medical records did not clearly document the reason for medication refusal. This may indicate that the patient either did not provide a reason for refusal, possibly because they were not asked for one, or their refusal was unrelated to the medication itself.

Among those who discontinued PP3M, 45.5% reverted back to PP1M and 21% of patients refused all medication. This unusually high level of all treatment refusal appears to be related to the loss of contact with patients that was previously provided by booked injection appointments. All of those who “refused” treatment simply failed to attend their next appointments after the last dose of PP3M, and contact was lost, thus emphasising the need to maintain contact with patients outside their depot injection schedule.

The second most common reason for discontinuation (24% of discontinuations for PP3M and 23% for PPLAIs) was the emergence of adverse effects. This is concerning given that all patients had previously been stabilised on PP1M and were presumably tolerating treatment well. Nonetheless, as a proportion of all those starting PP3M, only 8% stopped because of (apparently newly) emergent adverse effects – a small proportion overall.

## Limitations

The main limitation of this study is its observational, naturalistic, and non-interventional design. As such, there was no control group or blinding. Another limitation is the fact that PP3M was administered as a maintenance treatment to patients already stabilised on PP1M. As a result, it could be anticipated that continuation and relapse rates would both be low. As already mentioned, out cohort may not be generalisable to other ethnic groups. Finally, our definition of relapse may not align with other naturalistic studies and might not capture all mental state deteriorations.

## Summary

The main finding from this study is that an assured drug delivery formulation such as PP3M given at the right dose and frequency to fully compliant patients can significantly reduce the risk of relapse. The absence of relapse in the last 18 months of our observation period suggests that most or all relapse in practice may at least in part be caused by a failure to afford adequate antipsychotic plasma levels either because of poor compliance with oral treatment or a suboptimal frequency of LAI administration.

## Supporting information

Clark et al. supplementary material Clark et al. supplementary material

## Supplementary material

The supplementary material for this article can be found at https://doi.org/10.1192/j.eurpsy.2024.13.
