# Supplementary material for: Clinical outcomes with paliperidone palmitate 3-monthly injection as monotherapy: observational 3-year follow-up of patients with schizophrenia
Source: Eur Psychiatry. 2024 Mar 7;67(1):e15. doi: 10.1192/j.eurpsy.2024.13 (PMC10966611; doi:10.1192/j.eurpsy.2024.13)
Supplement: Clark et al. supplementary material [file S0924933824000130sup001.docx]

**Supplementary material**

***Table S1.*** *Baseline characteristics*

| Characteristic | Total | |
| --- | --- | --- |
|  | n= 111 | |
| *Age (years)* | | |
| Mean, SD | 43.2, 11.2 | |
| Min, max | 20, 65 | |
| *Prior duration of PP1M (months)* | | |
| Mean, SD | 33.6, 24.9 | |
| Min, max | 3, 85 | |
|  | n (%) | |
| *Sex* | | |
| Female | 35 | (31.5) |
| Male | 76 | (68.5) |
| *Ethnicity* | | |
| Asian/ Asian British^1^ | 7 | (6.3) |
| Black/ Black British | 72 | (64.9) |
| Mixed Background | 5 | (4.5) |
| Other | 8 | (7.2) |
| White | 19 | (17.1) |
| *Care setting on initiation of PPLAI (1M)* | | |
| Inpatient | 57 | (51.4) |
| Outpatient | 54 | (48.6) |
| *Considered treatment-responsive* | | |
| No | 34 | (30.6) |
| Yes | 77 | (69.4) |
| *Polypharmacy for ≥1 month* | | |
| No^2^ | **97** | **(87.4)** |
| Yes | **14** | **(12.6)** |
| Oral aripiprazole | 5 | (4.5) |
| Oral olanzapine | 2 | (1.8) |
| Oral risperidone | 6 | (5.4) |
| Oral aripiprazole and oral risperidone | 1 | (0.9) |
| *Prior PP1M dose* | | |
| 150mg | 39 | (35.1) |
| 100mg | 44 | (39.6) |
| 75mg | 18 | (16.2) |
| 50mg | 10 | (9.0) |

*^1^Asian refers to Indian-subcontinent as per NHS ethnicity classifications.*

*^2^One patient received one administration of flupentixol 100mg in error.*

***Table S2****. PP3M characteristics*

| PP3M | Total | |
| --- | --- | --- |
|  | n= 111 | |
| *Initial PP3M dose* [n (%)] |  | |
| 525mg | 36 | (32.4) |
| 350mg | 46 | (41.4) |
| 263mg | 18 | (16.2) |
| 175mg | 11 | (9.9) |
| *Dose change* [n (%)] |  | |
| None | **87** | **(85.3)** |
| Increase [n (%)] | **4** | **(3.9)** |
| Worsening symptoms | 3 | (75.0) |
| Patient request | 1 | (25.0) |
| Decrease [n (%)] | **11** | **(10.8)** |
| Patient request | 5 | (45.5) |
| Adverse effects | 4 | (36.4) |
| Improved condition | 1 | (9.1) |
| Unclear | 1 | (9.1) |

***Table S3*** *Substance abuse*

| *Substance abuse (SA)* | Total | |
| --- | --- | --- |
|  | n=111 | |
|  | n | % |
| No history of SA or confirmed diagnosis | **40** | **(36.0)** |
| History of SA | **61** | **(55.0)** |
| Diagnosis of SA | **10** | **(9.0)** |
| F10 | 1 | (1) |
| F12 | 5 | (4.5) |
| F19 | 3 | (3) |
| F10 and F19 | 1 | (1) |
| *Continuers* | 62 |  |
| No history of SA or confirmed diagnosis | 27 | (43.5) |
| History of SA | 33 | (53.2) |
| Diagnosis of SA | 2 | (3.2) |
| *Discontinuers* | 40 |  |
| No history of SA or confirmed diagnosis | 11 | (27.5) |
| History of SA | 23 | (57.5) |
| Diagnosis of SA | 6 | (15.0) |
| *Relative risk (RR) 95%CI* | | |
| Discontinuation and SA | 1.28 | 0.961- 1.717 |


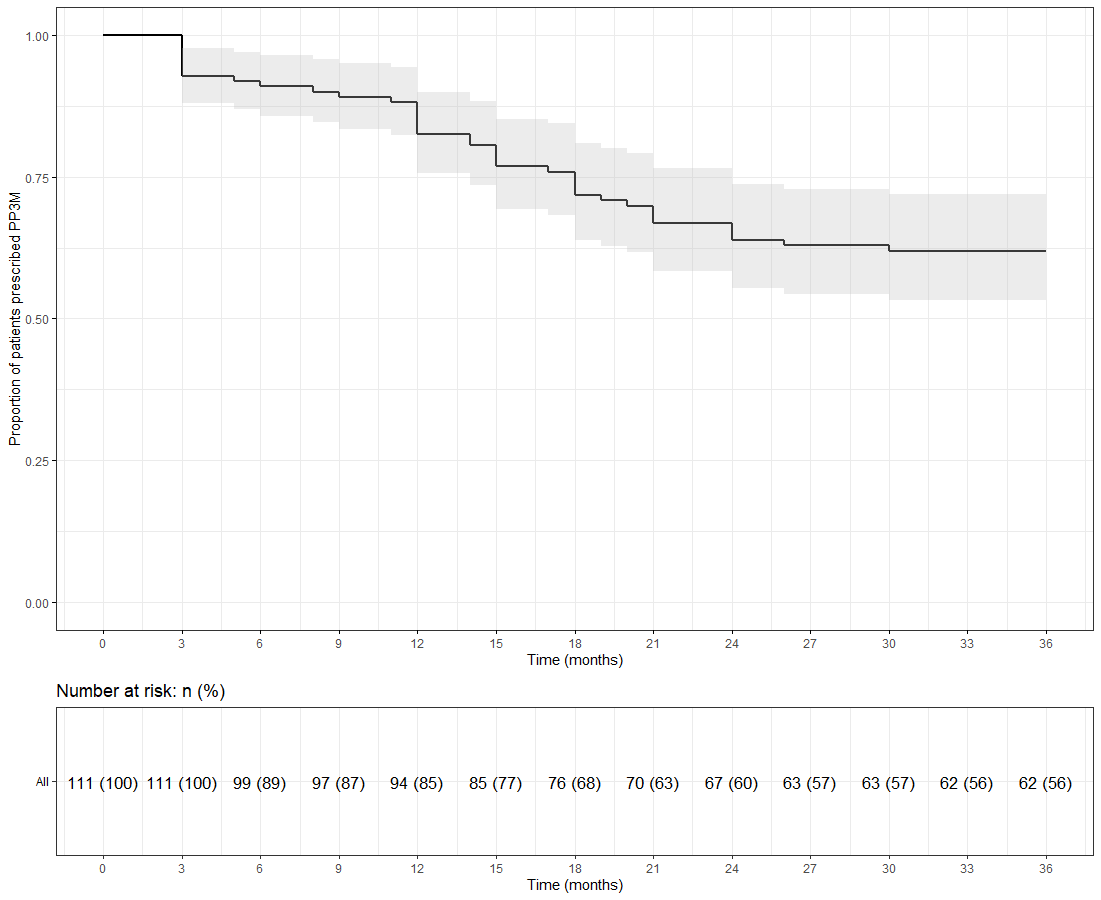


*Figure S1: Kaplan-Meier plot showing the proportion of patients prescribed PP3M since initiation and at 36 months*

***Table S4.*** *Discontinuation from PP3M*

| Clinical Outcome after three years (PP3M) |  | |
| --- | --- | --- |
|  | n= 111 | |
| *Outcome three years after initiating PP3M* [n (%)] | | |
| Continuation | 62 | (55.9) |
| Discontinuation | 40 | (36.0) |
| Attrition | 9 | (8.1) |
|  | n= 40 | |
| *Reasons for discontinuation* [n (%)] |  | |
| Patient refusal | 12 | (30.0) |
| Perceived inefficacy | 9 | (22.5) |
| Adverse effects | 9 | (22.5) |
| Patient request | 5 | (12.5) |
| Independent health condition^1^ | 3 | (7.5) |
| Need more flexible dose adjustment | 2 | (5.0) |
| *Next medication (6 months after last depot)* [n (%)] |  | |
| PP1M | 19 | (47.5) |
| No medication* | 7 | (17.5) |
| Risperidone (oral) | 4 | (10.0) |
| Aripiprazole (oral) | 4 | (10.0) |
| Haloperidol (oral) | 2 | (5.0) |
| Quetiapine (oral) | 1 | (2.5) |
| Flupentixol (LAI) | 1 | (2.5) |
| Zuclopenthixol (LAI) | 1 | (2.5) |
| Lurasidone (oral) | 1 | (2.5) |
|  | n= 9 | |
| *Reasons for attrition* [n (%)] |  | |
| Lost to follow-up^2^ | 7 | (77.8) |
| Died^3^ | 2 | (22.2) |

^1^Independent health condition: cancer (n=2) and kidney problems.

^2^Lost to follow up: missing person (n=3), left country (n=2), changed trust (n=1), disengagement (n=1)

^3^Deaths classified using ePJS and not at a systems level: non-adherence to diabetes medication (n=1)

and unknown (n=1).

*All refused medication.

***Table S5.*** *Discontinuation from PPLAI (1M or 3M)*

| Clinical Outcome after three years (1M or 3M) |  | |
| --- | --- | --- |
|  | n= 111 | |
| *Outcome three years after initiating PPLAIs (1M and 3M)* [n (%)] | | |
| Continuation | 74 | (66.7) |
| Discontinuation | 26 | (23.4) |
| Attrition | 11 | (9.9) |
|  | n= 25 | |
| *Reasons for discontinuation* | [n (%)] | |
| Patient refusal | 10 | (43.5) |
| Adverse effects | 5 | (21.7) |
| Perceived inefficacy | 3 | (13.0) |
| Patient request | 3 | (13.0) |
| Independent health condition | 2 | (8.7) |
|  | n= 11 | |
| *Reasons for attrition* [n (%)] |  | |
| Lost to follow-up^1^ | 9 | (81.8) |
| Died | 2 | (18.2) |

*^1^ The two additional patients lost to follow-up were because of changing trust and being discharged to their GP.*


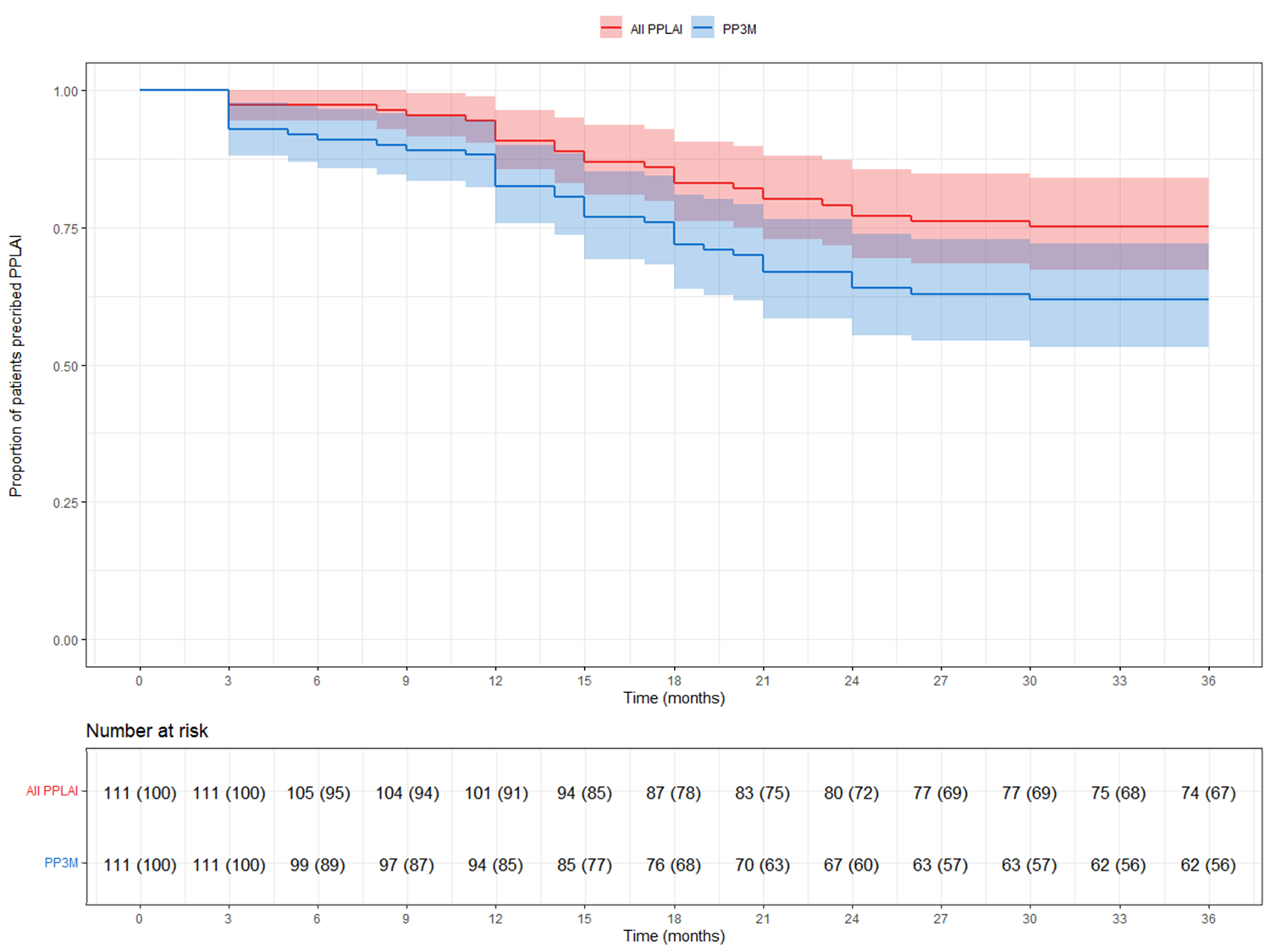


*Figure S2- Kaplan-Meier plot showing the proportion of patients (with 95% confidence interval) prescribed PPLAI since PP3M initiation. All PPLAI (PP3M followed by PP1M and in some cases PP3M again) is shown in red and PP3M only is shown in blue.*


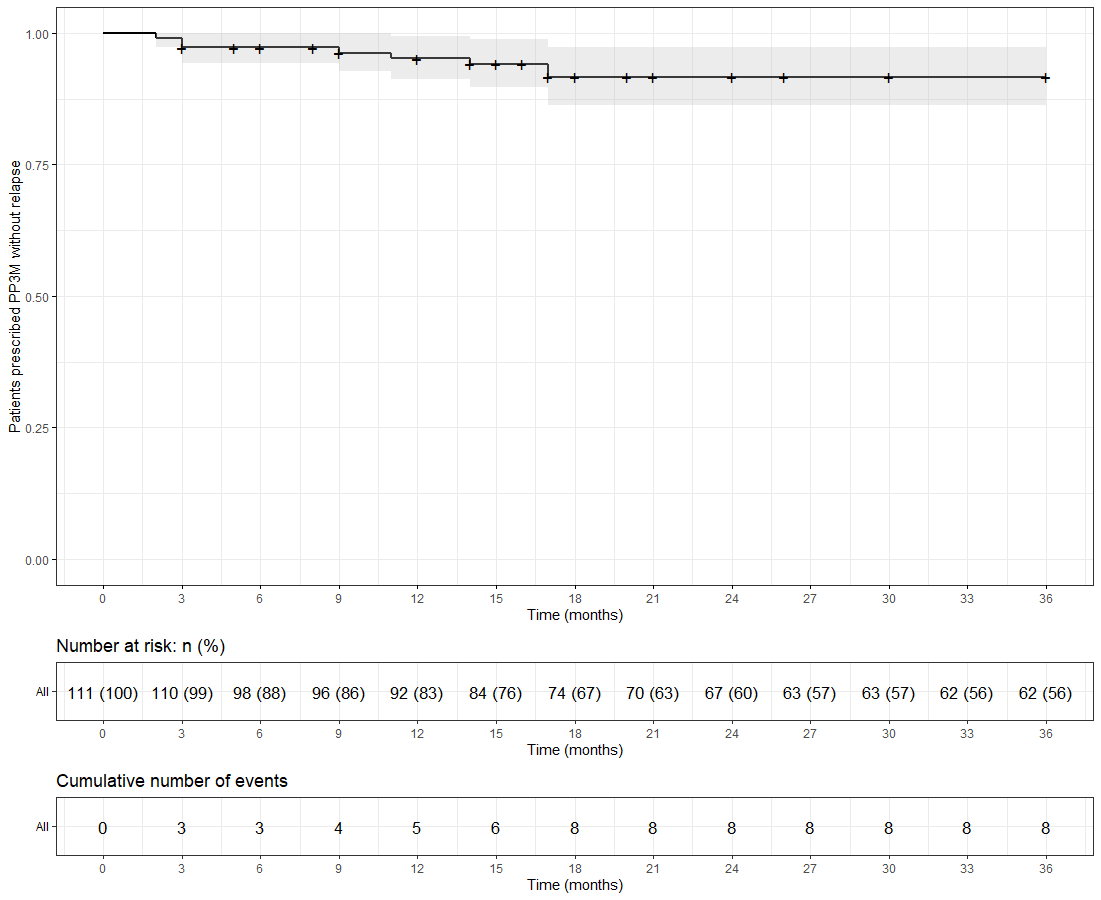


*Figure S3- Kaplan-Meier plot showing the proportion of patients that relapsed (with 95% confidence interval) whilst being prescribed PP3M. Patients who discontinued were censored (shown as a dash on the plot).*


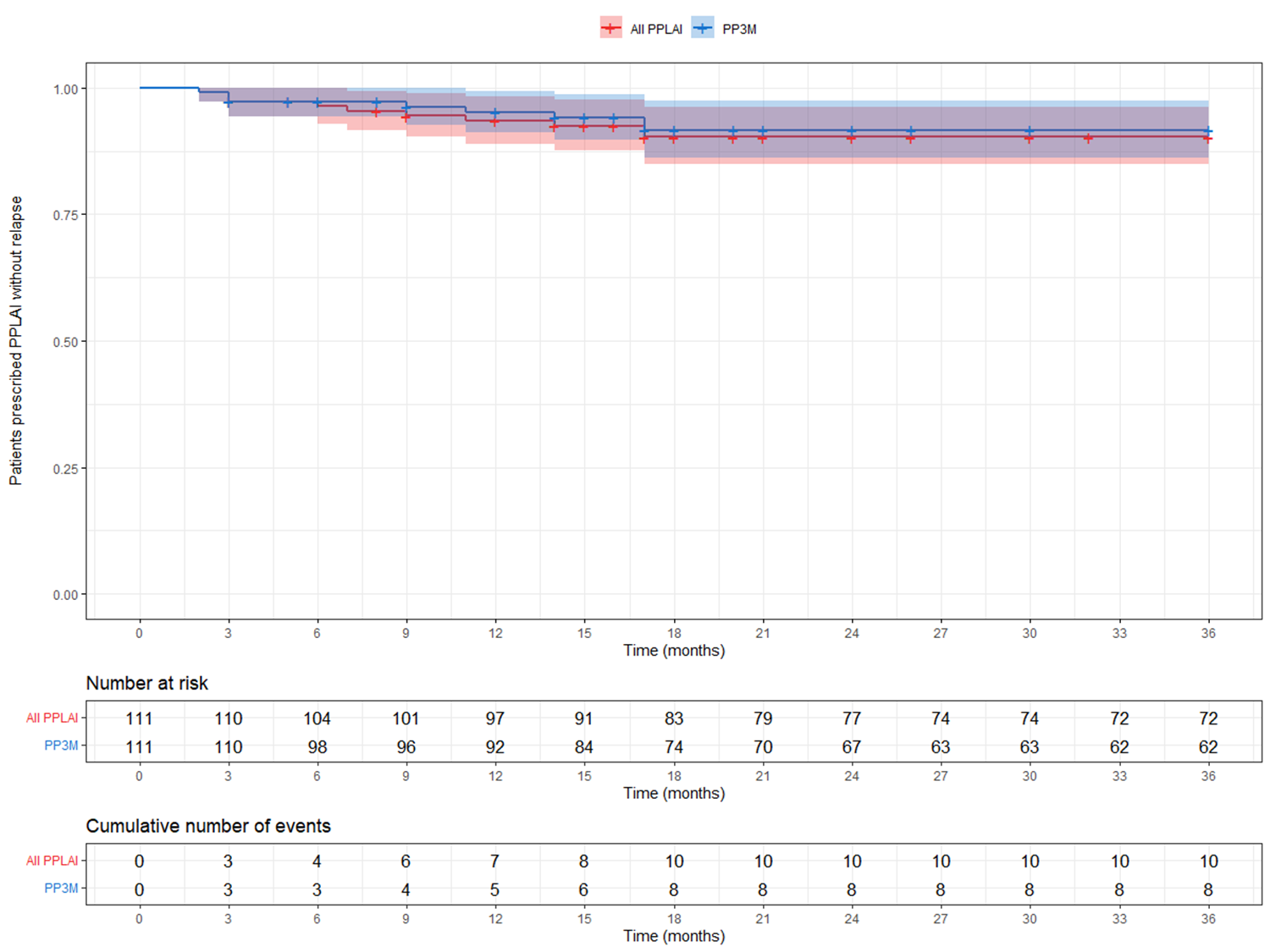


*Figure S4- Kaplan-Meier plot showing the proportion of patients that relapsed (with 95% confidence interval) whilst being prescribed PPLAI. Patients who discontinued were censored (shown as a dash on the plot). All PPLAI (PP3M followed by PP1M and in some cases PP3M) is shown in red and PP3M only is shown in blue*.
